# Identification of Inappropriately Reprogrammed Genes by Large-Scale Transcriptome Analysis of Individual Cloned Mouse Blastocysts

**DOI:** 10.1371/journal.pone.0011274

**Published:** 2010-06-30

**Authors:** Atsushi Fukuda, Feng Cao, Shinnosuke Morita, Kaori Yamada, Yuko Jincho, Shouji Tane, Yusuke Sotomaru, Tomohiro Kono

**Affiliations:** 1 Department of Bioscience, Tokyo University of Agriculture, Tokyo, Japan; 2 Natural Science Centre for Basic Research and Development, Hiroshima University, Hiroshima, Japan; CNRS, France

## Abstract

Although cloned embryos generated by somatic/embryonic stem cell nuclear transfer (SECNT) certainly give rise to viable individuals, they can often undergo embryonic arrest at any stage of embryogenesis, leading to diverse morphological abnormalities. In an effort to gain further insights into reprogramming and the properties of SECNT embryos, we performed a large-scale gene expression profiling of 87 single blastocysts using GeneChip microarrays. Sertoli cells, cumulus cells, and embryonic stem cells were used as donor cells. The gene expression profiles of 87 blastocysts were subjected to microarray analysis. Using principal component analysis and hierarchical clustering, the gene expression profiles were clearly classified into 3 clusters corresponding to the type of donor cell. The results revealed that each type of SECNT embryo had a unique gene expression profile that was strictly dependent upon the type of donor cells, although there was considerable variation among the individual profiles within each group. This suggests that the reprogramming process is distinct for embryos cloned from different types of donor cells. Furthermore, on the basis of the results of comparison analysis, we identified 35 genes that were inappropriately reprogrammed in most of the SECNT embryos; our findings demonstrated that some of these genes, such as *Asz1*, *Xlr3a* and *App*, were appropriately reprogrammed only in the embryos with a transcriptional profile that was the closest to that of the controls. Our findings provide a framework to further understand the reprogramming in SECNT embryos.

## Introduction

Since the birth of “Dolly,” the first mammal to be cloned from somatic cells in 1997, extensive efforts have been made to understand the mechanisms that underlie the reprogramming of the donor cell genome after its transplantation into recipient oocytes [1], [2]. Despite these efforts, researchers have been unable to elucidate the molecular mechanisms underlying this phenomenon, whereby genes are silenced or activated by epigenetic DNA modification or by binding of certain proteins to the donor cell genome. This process is undoubtedly influenced by factors that are specific to metaphase II (MII) oocytes or mitotic zygotes [3], which induces the most dynamic transition from the terminally differentiated state of the genome to the totipotent one. This dynamic transition in developmental reprogramming is greater than that observed in the generations of induced pluripotent stem cells (iPSCs) [4], [5], [6], [7], [8]. Therefore, an understanding of the mechanism underlying nuclear reprogramming in cloning will certainly contribute toward the advancement of therapeutic stem cell technology [9], [10], [11], [12].

The complete reprogramming required for normal development is induced in only a few cases; consequently, faulty epigenetic changes accompanied by diverse abnormalities in the development of somatic/embryonic stem cell nuclear transferred (SECNT) embryos occur very frequently [13], [14], [15], [16], [17], [18]. Research on pre- and postimplantation development of SECNT embryos has shown that the embryos rapidly lose their developmental ability around the time of implantation, resulting in failure of implantation and normal embryogenesis [19], [20]. These results indicate that the faulty epigenetic changes occur at the preimplantation stage. Our previous study revealed that 60% or more of mouse embryos cloned from embryonic stem (ES) cells developed into blastocysts; however, less than 10% of these blastocysts resulted in E9.5 fetuses [20], [21]. Even if a SECNT embryo survives, permanent adverse effects are often manifested as abnormalities such as large-offspring syndrome, placental enlargement, adiposity, respiratory defects, and immune defects, all of which result in a shortened lifespan [22], [23], [24], [25], [26]. Interestingly, the abnormal phenotypes observed in cloned mice were restored in their offspring, suggesting that the epigenetic failure was normalized in the germ line [26], [27].

Recent studies directed at improving our understanding of the properties of SECNT embryos are based on transcriptome analysis using oligo microarrays [28], [29], [30], [31] and cDNA subtraction [20]. However, because most of these studies were performed using pooled embryo samples, the results are often unclear and difficult to interpret. In the case of pooled samples, differentially expressed genes are screened on the basis of their mean expression levels, whereby some genes that are truly differentially expressed in SECNT embryos may not be detected. This problem is further compounded by the fact that SECNT embryos display a marked degree of heterogenecity in their gene expression profiles [32]. In addition, many studies have been conducted to evaluate the reprogramming of epigenetic processes such as DNA methylation and histone acetylation/methylation [13], [33], [34], [35], [36]. The results of these studies have improved to some extent our understanding of reprogramming; however, similar to the results of transcriptome analysis, these findings often reflect either the global but nonspecific changes or the local but specific changes occurring due to reprogramming.

Transcriptome analysis of individual embryos is indispensable for gaining a deeper insight into the molecular mechanisms underlying the reprogramming of donor nuclei in SECNT embryos. In an effort to elucidate the novel and genuine properties of SECNT embryos, we conducted a large-scale transcriptome analysis of single SECNT blastocysts using oligo microarrays. The SECNT embryos were reconstructed using Sertoli (SR) cells, cumulus (CU) cells, and ES cells. Gene network and canonical pathway analyses revealed specific functional disorders occurring in SECNT embryos. Furthermore, by systematic comparison of the gene expression profiles of individual blastocysts, we were able to identify truly differentially expressed genes in the SECNT embryos. The present study is the first to evaluate the properties of individual SECNT embryos using transcriptomic profiles—an approach that can help decipher the mechanism of reprogramming.

## Results and Discussion

### Genes Differentially Expressed Between Cloned and Control Blastocysts

**Figure 1:**
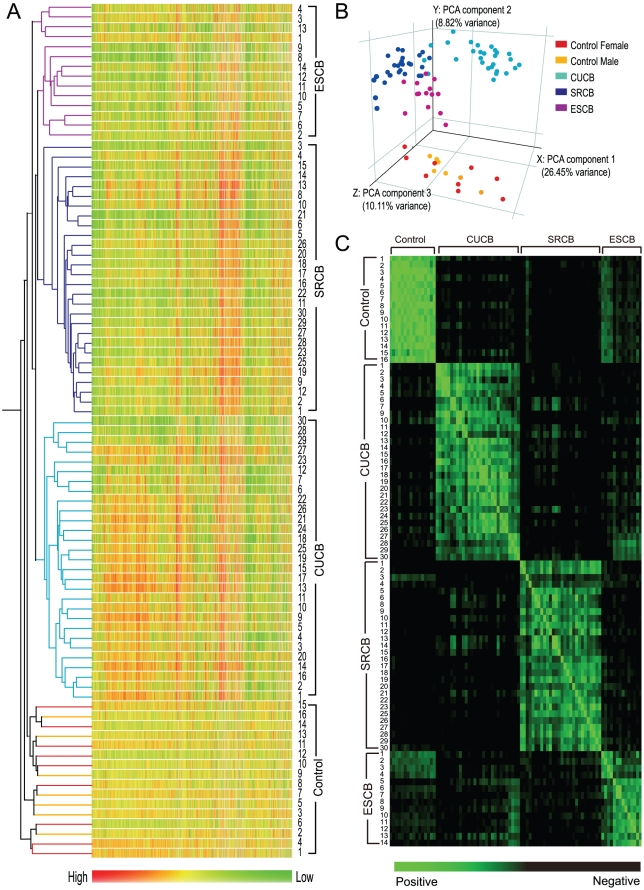
Gene Expression Profile Analysis of Individual Blastocysts. (A) Hierarchical clustering of all the SECNT samples. CUCBs: n = 29; SRCBs: n = 28; ESCBs: n = 14, and control blastocysts: n = 16. Colors correspond to the relative RNA abundance for more than 39,000 transcripts. Numbers marked beside each profile are individual figures of samples. (B) Principal component analysis of gene expression in all the samples subjected to the hierarchical clustering analysis. (C) Correlation matrix based on the Pearson coefficient of correlation between 2 corresponding samples. The correlation between samples is shown by a color scale ranging from green (positive correlation) to black (negative correlation). The coefficients of correlation are provided in Table S4.

In order to evaluate the reprogramming status at the blastocyst stage, we performed oligo microarray analysis of 87 blastocysts including those derived from CU cells (CUCBs; n = 29), those derived from SR cells (SRCBs; n = 28), those derived from ES cells (ESCBs; n = 14), and control blastocysts (n = 16). Using the data obtained by GeneChip 430 2.0 microarray analysis, we performed hierarchical clustering using the GeneSpring GX7.3 software and constructed a dendrogram for the 87 samples (Figure 1A). The analysis clearly showed that the gene expression profiles of the SECNT embryos (71 samples) were invariably clustered into 3 groups corresponding to the type of donor cell used: the CUCB, SRCB, and ESCB groups. In clustering analysis, the profiles of the ESCBs were placed close to those of the controls. This indicates the similarity between the gene expression profiles of the 2 groups; however, we have already confirmed that embryos cloned from ES cells lack the ability to develop to term [20].

The validity of this clustering was supported by principal component analysis (PCA; x-axis, PCA component 1: 26.45% variance; y-axis, PCA component 2: 8.82% variance; and z-axis, PCA component 3: 10.11% variance), which was performed using the 17,747 probe sets selected after GeneSpring normalization (Figure 1B). The cloned embryos were separated from the control embryos most obviously by the second principal component (y-axis), which had a variance of 8.82%. This means that approximately 1,460 of the total 17,747 probe sets analyzed in this experiment may be determinative factors, which are worthy of further attention. The concordance between the results from the hierarchical cluster analysis and PCA indicated that the global gene expression pattern of the SECNT embryos was different from that of the controls and that the profiles of the cloned embryos clearly formed 3 clusters corresponding to each donor cell type. Thus, the present study represents the first large-scale and high-quality transcriptome analysis in individual preimplantation stage embryos. Although a few studies on microarray analysis of individual preimplantation embryos of cattle have been reported [30], [31], the results are not entirely satisfactory because they are based on microarray analyses using a small number of probe sets or because only a limited number of analyses were performed.

Interestingly, the gene expression profiles of 2 SRCBs, SR3 and SR4, were remarkably similar to those of the control blastocysts (Figure 1A). These profiles were clearly distinguishable from those of CUCBs and ESCBs as well as other SRCBs. In order to verify this finding, we constructed a correlation matrix for comparing the Pearson coefficient of correlation between the controls and cloned embryos (Figure 1C and Table S1). It showed that only the gene expression profile of SR3 differs significantly from that of other SRCBs. This finding was supported by the fact that SRCBs were able to develop to term at the efficiency of approximately 4.2% (Table S2), with the expected frequency for viable individuals developing from 28 SRCBs being 1.176. This suggests that reprogramming had been successful in the case of at least 1 SRCB and that this embryo would have acquired the competency to develop to term. Therefore, for a more accurate analysis of SRCBs, we conducted the subsequent analysis by excluding the data for SR3 and SR4. A detailed description of the results regarding the gene expression profiles of SR3 will be provided later.

### Functional annotation analysis

**Figure 2:**
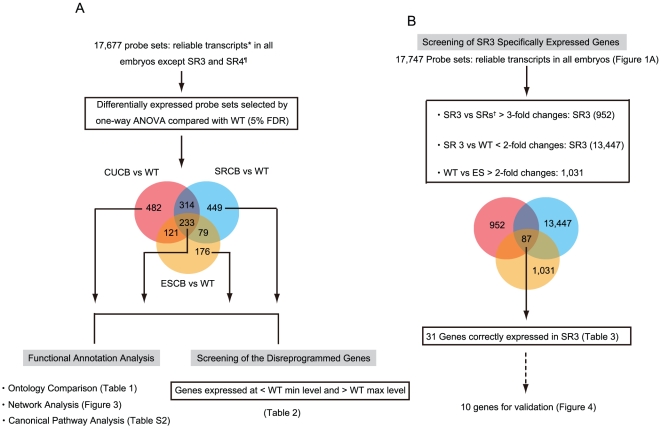
Flow Chart Depicting the Screening of Inappropriately Reprogrammed and SR3-specific Genes. *: > raw signal intensity value of 100 for at least 1 embryo. (A) ¶: The number of probe sets was obtained from the all microarray data, excluding those for SR3 and SR4, because the gene expression profiles of these embryos were remarkably similar to those of the controls (Figure 1A). (B) †: SRs indicates all embryos cloned from SR cells, except SR3 and SR4.

One-way analysis of variance (ANOVA) was performed with the post-hoc test at a false discovery rate of 5%, after GeneSpring normalization. The results of the analysis showed that a relatively large number of probe sets were significantly differentially expressed between the controls and the 3 SECNT embryo groups: 1,150 (upregulated 531; downregulated 619 [53.83%]), 1,075 (upregulated 565; downregulated 510 [47.44%]), and 609 (upregulated 180; downregulated 429 [70.44%]), in CUCB, SRCB, and ESCB groups, respectively (Figure S1). Next, on the basis of the mean expression value data, we constructed a Venn diagram for affiliation analysis (Figure 2A). This diagram showed that 233 probe sets were common to all SECNT embryo groups (ALL). The numbers of probe sets specifically expressed in each of the SECNT embryo groups were as follows: CUCB, 482; SRCB, 449; and ESCB, 176. The gene expression profiles of the ESCBs appeared to resemble those of the control embryos; however, our previous study showed that no viable individual could be generated from ES cells [20].

In order to understand the biological roles of the differentially expressed genes, we used FatiGO at Babelomics (www.fatigo.org) for ontological comparison of the probe sets segregated using the post-hoc test (Figure 2A). According to the data on statistical significance (P<0.05), the top gene ontology (GO) categories defined on the basis of the probe sets differentially expressed in each blastocyst group were associated with the following: the probe sets of ALL and CUCBs were associated with transferase activity; those of SRCBs, with sterol biosynthetic process; and those of regulation of biological process, with ESCBs. The other functions of each cloned group are described in Table 1. To better understand the causes of specific disorders in each cloned group, we compared each donor cells with respect to the expression levels of the genes that were placed in the top GO categories; the data on the donor cells were obtained by microarray analysis (CBX109). Interestingly, from the 151 genes that were involoved in transferase activity in CUCBs, 12 and 7 genes were expressed at low and high levels, respectively, in CU cells at threefold or more. From the 17 genes that were involoved in the sterol biosynthetic process in SRCBs, 3 genes showed expression at threefold < in SR cells. Of the 154 genes that were involoved in regulation of biological process in ESCBs, 12 and 7 genes were expressed at low and high levels, respectively, in ES cells at threefold or more (Table S3). These results support the hypothesis that the level of gene expression in the donor cells is responsible for disorders pertaining to specific biological functions in SECNT embryos.

**Table 1:** Gene Ontology Analysis.

	All	CU	SR	ES
**1**	transferase activity	transferase activity	sterol biosynthetic process	regulation of biological process
**2**	positive regulation of cellular process	nucleotide binding	cholesterol biosynthetic process	primary metabolic process
**3**	death	transferase activity, transferring phosphorus-containing groups	sterol metabolic process	anatomical structure development
**4**	reproductive process	purine nucleotide binding	lipid metabolic process	cell cycle
**5**	anatomical structure development	ATP binding	cholesterol metabolic process	cellular metabolic process
**6**	positive regulation of biological process	sterol metabolic process	steroid biosynthetic process	response to endogenous stimulus
**7**	regulation of a molecular function	kinase activity	steroid metabolic process	macromolecule metabolic process
**8**	organismal movement	biopolymer modification	alcohol metabolic process	cellular developmental process
**9**	cell adhesion	adenyl nucleotide binding	amino acid biosynthetic process	regulation of a molecular function
**10**	biosynthetic process	phosphotransferase activity, alcohol group as acceptor	lipid biosynthetic process	cellular component organization and biogenesis

Gene Ontology catalog was ordered by adjusted p-value returned by FatiGO.

**Figure 3:**
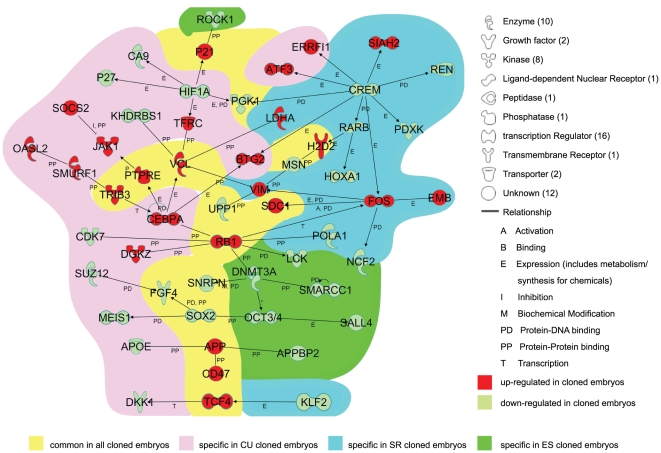
Network of Differentially Expressed Genes. The network was constructed by direct connection only. Background color represent the genes differentially expressed in all cloned embryos (yellow), CUCBs (pink), SRCBs (blue) and ESCBs (green). The red-colored symbols represent the genes upregulated in the cloned embryos and the light-green-colored symbols represent the downregulated ones.

In order to gain further insight into the mechanisms responsible for developmental arrest in each type of SECNT embryo, we performed an Ingenuity Pathways Analysis (IPA) using the list of differentially expressed genes. The results of the analysis showed that the networks of the differentially expressed genes reflected the embryonic origin of the genes (Figure 3). Moreover, to identify the genes involved in the fundamentally and biologically specific disorders in each cloned group, we performed Canonical pathway analysis using the list of differentially expressed genes. Of the several pathways shown in Table S4, some are known to lead to embryonic lethality. Considering the data on statistical significance and the relevance of the genes to pluripotency, embryonic development, and cell proliferation, we focused on some molecules in the networks, and our findings are discussed in further details in the following sections.

### Common to All Cloned Groups

In mice, pluripotency-associated genes play an important role in embryogenesis. Some of the genes downregulated in the clones were associated with pluripotency (Figure 3). For example, null mutation of *Sox2* and *Fgf4*, which were repressed in all the 3 groups, is known to cause embryonic lethality after implantation [37], [38]. In ES cells, the expression levels of these genes are strictly regulated in order to maintain the pluripotency; therefore, the disruption of poise in the genes expression levels lead to the loss of pluripotency [39]. Consequently, although the genes associated with pluripotency could be reactivated in most of the cloned embryos, inadequate expression levels of the pluripotency-associated genes might be partly responsible for the rapid embryo loss after implantation.

On the other hand, almost all the upregulated genes were related to the immune system and cell cycle. The genes related with the cell cycle merit further investigation because the cell cycle in mammalian embryonic cells at early stages of development differs greatly from that of somatic cells [40]. Interestingly, the gene retinoblastoma (*Rb*), which is responsible for a major G1 checkpoint and blocks S-phase entry and cell growth [41], was overexpressed in all the 3 groups. In somatic cells, mitogenic factors exert their effects on cell cycle progression via *Rb*-mediated pathways [40], while in mouse embryonic cells at the early cleavage stage, which is characterized by a short G1 phase, *Rb* expression is repressed until the blastocyst stage [42]. Interestingly, the developmental capacity to blastocyst stage was strikingly reduced in mouse embryos with overexpression of *Rb* [42]. Therefore, overexpression of *Rb* may be involved in the limited developmental capacity of SECNT embryos, but the exact role of *Rb* in the preimplantation stages of development of mice embryos has not yet been clearly defined. To obtain more detail information on faulty reprogramming occurring in cloned embryos, it would be necessary to scrutinize the global gene expression pattern before the blastocyst stage.

p21Cip1, a cell-cycle-dependent kinase inhibitor, was also overexpressed in the 3 groups, suggesting that the impaired development of cloned embryos may be caused by the deregulation of p21Cip1 because the upregulation of p21Cip1 arrests cell growth in the G1 phase [43].

Recently, the upregulation of p16Ink4a, p19Arf, p21Cip1, and p53 (the feature of senescence) has been shown to impede the reprogramming of somatic cells into iPSCs [44].

### CUCBs

In CUCB-specific gene networks, most of the genes encoded transcriptional regulators. Unexpectedly, there was no pluripotency-associated gene in these networks. Of these genes, *Suz12*, is essential for embryonic development; this gene encodes one of the components of the polycomb repressive complex 2 (PRC2), which catalyzes the di- and trimethylation of H3K27 [45]. Recent studies revealed that PRC2 was repressed and that H3K27me3 modification was absent in the inner cell mass (ICM) of cloned blastocysts [46]. In this study, we also identified *Suz12* as a differentially expressed gene, suggesting that the downregulation of *Suz12* could possibly lead to serious consequences due to its intrinsic ability to affect global histone modification.

Among the upregulated genes, the gene encoding myeloid transcription factor CCAAT/enhancer-binding protein-α (Cebpα) is a powerful inhibitor of cell proliferation [47]. Although studies have shown that the proliferation-inhibitory activity of Cebpα is not related to Rb, Cebpα activates p21Cip1, which is overexpressed in CUCBs, to suppress cell proliferation [47]. This suggests that the upregulation of Cebpα, but not p53, might trigger the disruption of p21Cip regulation.

### SRCBs

The SRCB-specific gene networks were constructed using the IPA database; however, the role of the genes comprising this network, except *Klf2*, in embryonic development are largely unknown. The deletion of the gene *Klf2* results in embryonic lethality between E12.5 and E14.5 due to circulatory defects [48]. Since the gene was significantly downregulated in SRCBs, it is suggested that the repression of this gene might contribute to disorders specific to SRCBs. On the other hand, these blastocysts overexpressed *Fos*, which induces the expression of *Rb* in HeLa cells [49]. This may indicate that the deregulation of *Rb* in SRCBs might be caused by the overexpression of *Fos*.

### ESCBs

The ESCB-specific gene network consisted of only downregulated genes. One of the most interesting features of this network was that the expressions of *Oct3/4*, which plays a critical role in maintaining the pluripotency in ICM [39], and *Fgf4* were downregulated in these blastocysts, whereas they were expressed in the donor ES cells (data not shown). However, *Cdx2*, which is expressed only in the trophectderm [50], showed normal expression levels. These finidngs indicate that ESCBs could not maintain the pluripotency, which might explain why none of these blastocysts developed into individuals.

Taken together, the deregulation of genes associated with pluripotency and the cell cycle in the 3 types of embryos used in this study lends credence to the notion that genes identified as differentially expressed between these embryos and the control embryos play a critical role in determining the developmental fate of cloning; the implications of these findings and need further investigation.

### Identification of Inappropriately Reprogrammed Genes in SECNT Embryos

Studies have suggested that inappropriate reprogramming occurs in a random manner for the majority of genes expressed in SECNT embryos [16], [17]. Currently, however, this notion has not been corroborated with sufficient and convincing evidence, and genes that are inappropriately reprogrammed in SECNT embryos have not yet been identified. Using microarray data, we were able to identify a particular set of genes that were inappropriately reprogrammed in each SECNT embryo group: 31 genes in CUCBs, 46 genes in SRCBs, and 11 in ESCBs (Table 2). Furthermore, we identified 35 genes from the 233 probe sets that were differentially expressed in all SECNT embryo groups. Of these 35 genes, 28 were downregulated in more than 62 (92%) of the SECNT embryos (Table 2). In contrast, only a limited number of genes were upregulated: *Cd81* in 67 cases, and *Cd47* and *Rbms1* in 66 cases each.

**Table 2:** Inappropriately Reprogrammed Genes.

**All (35**	**genes)**	**up-7**		*Dctd*	8	100%	DCMP deaminase	*Tnfrsf10b*	14	100%	tumor necrosis factor receptor superfamily, member 10b
**Gene name**	**Chr**	**Reliability**	**Discription**	*Stk31*	6	(29/29)	Serine threonine kinase 31	*3110003A17Rik*	10	(26/26)	RIKEN cDNA 3110003A17 gene
*Cd81*	7	97% (67/69)*	CD 81 antigen	*Ly6e*	15		Lymphocyte antigen 6 complex, locus E	*Hspa1b*	17		heat shock protein 1B
*Cd47*	16	96% (66/69)	CD47 antigen (Rh-related antigen, integrin-associated signal transducer)	*Baiap2l1*	5		BAI1-associated protein 2-like 1	*Hmgcr* **	13		3-hydroxy-3-methylglutaryl-Coenzyme A reductase
*Rbms1*	2		RNA binding motif, single stranded interacting protein 1	*Spg20*	3		Spastic paraplegia 20, spartin (Troyer syndrome) homolog (human)	*Cklfsf8* **	9		CKLF-like MARVEL transmembrane domain containing 8
*Seh1l*	18	94% (65/69)	SEH1-like (S. cerevisiae)	*H2-Bl*	17		Histocompatibility 2, blastocyst	*Slc25a1* **	16		solute carrier family 25 (mitochondrial carrier, citrate transporter), member 1
*Mvd*	8	93% (64/69)	mevalonate (diphospho) decarboxylase	*H-2D1*	17		histocompatibility 2, D region locus 1	*Glipr2*	4		GLI pathogenesis-related 2
*Aga*	8	92% (63/69)	aspartylglucosaminidase	*Aurkc*	7		Aurora kinase C	*Camta1*	4		calmodulin binding transcription activator 1
*App*	16		amyloid beta (A4) precursor protein	*Ceacam10*	7		CEA-related cell adhesion molecule 10	*Stfa1*	16		stefin A1
**All (35**	**genes)**	**down-28**		*EG240038*	17		Predicted gene, EG240038	*Upk1b*	16		uroplakin 1B
**Gene name**	**chr**	**Reliability**	**Discription**	*D8Ertd67e*	8		DNA segment, Chr 8, ERATO Doi 67, expressed	*Gpr177* **	3		RIKEN cDNA 5031439A09 gene
*Asz1*	6	99% (68/69)	ankyrin repeat, SAM and basic leucine zipper domain containing 1	*Oas1f*	5		2′-5′ oligoadenylate synthetase 1F	*Alcam*	16		activated leukocyte cell adhesion molecule
*Nsbp1*	x		nucleosome binding protein 1	*Gm817*	1		Gene model 817, (NCBI)	*Rhou*	8		ras homolog gene family, member U
*Wnk1*	6		protein kinase, lysine deficient 1	*Ccdc112*			coiled-coil domain containing 112	*Timp3*	10		tissue inhibitor of metalloproteinase 3
*Zfp820*	17		zinc finger protein 820	*Ergic1*	17		Endoplasmic reticulum-golgi intermediate compartment (ERGIC) 1	*Spsb1* **	4		splA/ryanodine receptor domain and SOCS box containing 1
*Xlr3a*	x	97% (67/69)	X-linked lymphocyte-regulated 3A	*Ard1b*	5		ARD1 homolog B (S. cerevisiae)	*Tpm5*	3		tropomyosin 3, gamma
*Xlr4b*	x		X-linked lymphocyte-regulated 4B	*D7Ertd715e*	7		DNA segment, Chr 7, ERATO Doi 715, expressed	*Ptk6*	2		PTK6 protein tyrosine kinase 6
*Xlr5d*	x		X-linked lymphocyte-regulated 5C	*Apoe*	7		Apolipoprotein E	*Idi1*	13		isopentenyl-diphosphate delta isomerase
*Fmr1nb*	x		RIKEN cDNA 3830422N12 gene	*Ccdc112*	18		Coiled-coil domain containing 112	*Sc5d* **	9		sterol-C5-desaturase (fungal ERG3, delta-5-desaturase) homolog (S. cerevisae)
*Gyk*	x		glycerol kinase	*2610305D13Rik*	4		RIKEN cDNA 2610305D13 gene	*Il1rn* **	2		interleukin 1 receptor antagonist
*Msn*	x		moesin	*2610030H06Rik*	X		RIKEN cDNA 2610030H06 gene	*Trim35*	14		tripartite motif-containing 35
*Hsd17b10*	x	96% (66/69)	hydroxyacyl-Coenzyme A dehydrogenase type II	*Zfp264*	7		Zinc finger protein 264	*Cdgap* **	16		Cdc42 GTPase-activating protein
*Magea5*	x		melanoma antigen, family A, 5	*Ttc39c*	18		tetratricopeptide repeat domain 39C	*Btbd9*	17		BTB (POZ) domain containing 9
*Znf624*	12	94% (65/69)	zinc finger protein 97	*Baiap2l1*	5		BAI1-associated protein 2-like 1	*Anxa3*	5		annexin A3
*Cds2*	2		CDP-diacylglycerol synthase (phosphatidate cytidylyltransferase) 2	*Cetn2*	X		Centrin 2	**SRCB (46**	**genes)**	**down-7**	
*St8sia1*	6		ST8 alpha-N-acetyl-neuraminide alpha-2,8-sialyltransferase 1	*Ssr1*	13		Signal sequence receptor, alpha	**Gene name**	**chr**	**Reliability**	**Discription**
*C77370*	x		C77386 Mouse 3.5-dpc blastocyst cDNA Mus musculus cDNA clone J0030C05 3′, mRNA sequence.	*Il2rg*	X		Interleukin 2 receptor, gamma chain	*galectin-6*	7	100%	lectin, galactose binding, soluble 6
*Rps6ka6*	x		ribosomal protein S6 kinase polypeptide 6	*A530057A03Rik*	2		RIKEN cDNA A530057A03 gene	*Rpl39l* **	16	(26/26)	RIKEN cDNA 4930517K11 gene
*Tktl1*	x		transketolase-like 1	**SRCB (46**	**genes)**	**up-39**		*Tsr2*	X		TSR2, 20S rRNA accumulation, homolog (S. cerevisiae)
*Foxp3*	x	93% (64/69)	forkhead box P3	**Gene name**	**chr**	**Reliability**	**Discription**	*Mageb16* **	X		melanoma antigen family B, 16
*Tmem158*	9		transmembrane protein 158	*Asapk* **	3	100%	3′-phosphoadenosine 5′-phosphosulfate synthase 1	*Pim1*	17		proviral integration site 1
*Stmn3*	2		stathmin-like 3	*Sqle* **	15	(26/26)*	squalene epoxidase	*Cds2* **	2		CDP-diacylglycerol synthase (phosphatidate cytidylyltransferase) 2
*Prom1*	5		prominin 1	*Sparc*	11		secreted acidic cysteine rich glycoprotein	*Smc6l1*	12		SMC6 structural maintenance of chromosomes 6-like 1 (yeast)
*Oas1g*	5		2′-5′ oligoadenylate synthetase 1G	*Mvd* **	18		mevalonate (diphospho) decarboxylase	**ESCB (11**	**genes)**	**down11**	
*BC023829*	x		cDNA sequence BC023829	*Pkd2*	5		polycystic kidney disease 2	**Gene name**	**chr**	**Reliability**	**Discription**
*Trap1a*	x		tumor rejection antigen P1A	*Rab15*	12		RAB15, member RAS oncogene family	*Bcl3*	7	100%	B-cell leukemia/lymphoma 3
*Pfkfb1*	x		6-phosphofructo-2-kinase/fructose-2,6-biphosphatase 1	*Tspan1* **	4		tetraspanin 1	*Cugbp2*	2	(14/14)	CUG triplet repeat, RNA binding protein 2
*Hemt1*	15	92% (63/69)	hematopoietic cell transcript 1	*Pltp*	2		phospholipid transfer protein	*Rin3*	12		Ras and Rab interactor 3
*Pgk1*	x		phosphoglycerate kinase 1	*Osp94* **	3		heat shock protein 4 like	*Ptprs*	17		Protein tyrosine phosphatase, receptor type, S
**CUCB (31**	**genes)**	**up-3**		*Gpa33*	1		glycoprotein A33 (transmembrane)	*Oct3/4*			POU domain, class 5, transcription factor 1
**Gene name**	**chr**	**Reliability**	**Discription**	*Nmb* **	7		neuromedin B	*Socs2*			suppressor of cytokine signaling 2
*Btbd9*	17	100% (29/29)*	BTB (POZ) domain containing 9	*Echdc1*	10		enoyl Coenzyme A hydratase domain containing 1	*Tsr2*	X		TSR2, 20S rRNA accumulation, homolog (S. cerevisiae)
*Ptk6*	2		PTK6 protein tyrosine kinase 6					*Cetn2*	X		Centrin 2
*St6galnac4*	2		ST6 (alpha-N-acetyl-neuraminyl-2,3-beta-galactosyl-1,3) -N-acetylgalactosaminide alpha-2,6-sialyltransferase 4	*Mta3*	17		metastasis associated 3	*Mllt7*	X		Transcribed locus
**CUCB (31**	**genes)**	**Down-28**		*Ldlr* **	9		low density lipoprotein receptor	*Ube2a*	X		Ubiquitin-conjugating enzyme E2A, RAD6 homolog (S. cerevisiae)
**Gene name**	**chr**	**Reliability**	**Discription**	*Tm7sf2* **	19		transmembrane 7 superfamily member 2	*Il2rg*	X		Interleukin 2 receptor, gamma chain

*(Number of embryos differentially expressed/Number of embryos tested).

**The gene whose expression is recovered in SR3.

On the basis of the expression levels of the genes determined by microarray analysis, only 7 of the inappropriately reprogrammed genes common to all SECNT embryo groups (*Asz1*, *Magea5*, *Magea3*, *Xlr3a*, *Xlr5c*, *Hemt1*, and *Tktl1*) were markedly repressed to less than 10% of the average expression levels in the controls. It remains to be determined why these genes were repressed to a greater extent than any other genes. However, *Asz1*, *Magea5*, and *Magea3*, were included in large organized chromatin K9 modifications (LOCKs), which is the region enriched with histone H3 lysine 9 dimethylation (H3K9me2, repressive histone mark). The occurrence of LOCKs is dependent of histone methyltrasferase G9a [51]. Recent studies have shown that the expression levels of *Asz1* was upregulated in G9a^−/−^ ES cells compared with that of wild type ES cells [52]. Moreover, it has been also reported that the inhibitory effect of G9a by the small molecule BIX-01294 could improve the efficiency of reprogramming toward iPSCs [53]. These suggest that the G9a inhibition using the small molecule may facilitate the reprogramming of markedly repressed genes in cloned embryos.

To date, various genes have been implicated in the developmental failure observed in SECNT embryos [14], [20], [54]. For example, null mutations of the *Oct4*, *Stat3*, and *Sox2* genes, which are known to be undifferentiated cell makers, are lethal before midgestation in mice [37], [55], [56]. Interestingly, most of the genes selected in this study as differentially expressed in the majority of SECNT embryos are novel, and their biological functions are unknown. This suggests that our large-scale gene expression profile analysis has identified genes that are actually responsible for developmental failure in SECNT embryos. These results clearly show that the properties of each group of SECNT embryos are unique, and they reinforce the idea that the reprogramming process occurs in a specific manner depending on the epigenetic status of donor cells. Thus, the findings obtained in this study helped elucidate mechanisms responsible for developmental disorders in each type of SECNT embryo.

### Characteristics of the most successfully reprogrammed cloned embryo

In SRCBs, SR3 was selected as the almost successfully reprogrammed embryo. Interestingly, of the 46 inappropriately reprogrammed genes in SRCB, 19 were expressed at a normal levels in SR3 (Table 2). Additionally, the genes *Asz1* and *Fmr1nb*, which were repressed in all SECNT embryos, were normally expressed in SR3 (Figure S2). These data support the idea that SR3 was the most successsfully reprogrammed cloned embryo and that SR3 would have acquired the high competency to develop to term.

To gain further insight into more preciously reprogrammed genes specific to SR3, we screened a set of genes to select 31 genes that were specifically expressed in SR3 (Figure 2B and Table 3). Of these 31 genes, 12 genes were downregulated and the others were upregulated in embryos cloned from SR cells. Interestingly, of the 12 downregulated genes, 10 were mapped to the X chromosome. On the other hand, all the genes that were upregulated in the embryos cloned form SR cells were mapped to autosomes. These results indicate that the active X chromosome in the donor cells is inactivated after nuclear transfer, because *Xist*, which is essential for X inactivation in cis [57], [58], is expressed in SRCBs (data not shown). It has been reported that biallelic expression of *Xist* in cumulus-cloned embryos begins from the 6–8 cell stages, indicating that abnormal reprogramming of *Xist* occurs irrespective of donor sex difference [57].Unfortunately, the developmental role in these 31 genes is largely unknown. Therefore, the functional analysis of these genes could provide further understanding of the developmental disorders in the SECNT embryos.

**Table 3:** Genes Specifically Expressed in SR3.

Up-regulated in SR cloned embryos			
Gene name	Chr	Reliability	Discription
*Celsr1*	15	26/26	cadherin EGF LAG seven-pass G-type receptor 1
*App*	16	(100%)	amyloid beta (A4) precursor protein
*Cyp51*	5		cytochrome P450, family 51
*Abcg2*	6		ATP-binding cassette, sub-family G (WHITE), member 2
*2310014L17Rik*	7		RIKEN cDNA 2310014L17 gene
*Trip6*	5		thyroid hormone receptor interactor 6
*Timp3*	10		tissue inhibitor of metalloproteinase 3
*Sc5d*	9		sterol-C5-desaturase (fungal ERG3, delta-5-desaturase) homolog (S. cerevisae)
*Tm7sf2*	19		transmembrane 7 superfamily member 2
*Pus7l*	15		pseudouridylate synthase 7 homolog (S. cerevisiae)-like
*Sema3b*	9	25/26	sema domain, immunoglobulin domain (Ig), short basic domain, secreted, (semaphorin) 3B
*Trim44*	2	(96%)	tripartite motif-containing 44
*Syt12*	19		synaptotagmin XII
*Tnfrsf10b*	14		tumor necrosis factor receptor superfamily, member 10b
*Anxa8*	14		annexin A8
*AU016853*	-	24/26	AU016853 Mouse two-cell stage embryo cDNA Mus musculus cDNA clone J0730G12 3′, mRNA sequence.
*Unknown*	-	(92%)	Transcribed sequence with strong similarity to protein sp:P00722 (E. coli) BGAL_ECOLI Beta-galactosidase
*Pank1*	19		pantothenate kinase 1
*Gpx3*	11		glutathione peroxidase 3

Down-regulated in SR cloned embryos			
Gene name	Chr	Reliability	Discription
*Asz1*	6		ankyrin repeat, SAM and basic leucine zipper domain containing 1
*Msn*	X	26/26	moesin
*Pls3*	X	(100%)	plastin 3 (T-isoform)
*Mageb16*	X		melanoma antigen family B, 16
*Fmr1nb*	X		fragile X mental retardation 1 neighbor
*Pgk1*	X		phosphoglycerate kinase 1
*Prom1*	5		prominin 1
*Xlr5c*	X	25/26	X-linked lymphocyte-regulated 5C
*Xlr3a*	X	(96%)	X-linked lymphocyte-regulated 3A
*Magea5*	X		melanoma antigen, family A, 5
*Tktl1*	X		transketolase-like 1
*Trap1a*	X	24/26 (92%)	tumor rejection antigen P1A

To date, pluripotency-associated genes such as *Oct3/4* and *Sox2* have been candidate markers for the selection of highly competent cloned embryos [59], [60]. However, these genes might not be suitable as markers of viable cloned embryos because most of the pluripotency-associated genes were reactivated and it remains to be validated whether they are specifically expressed only in viable cloned embryos. In this study, judging from large-scale transcriptome analysis of individual cloned embryos, we identified 31 genes that may be strong candidates for markers of highly competent cloned blastocysts.

### Validation of Microarray Data

**Figure 4:**
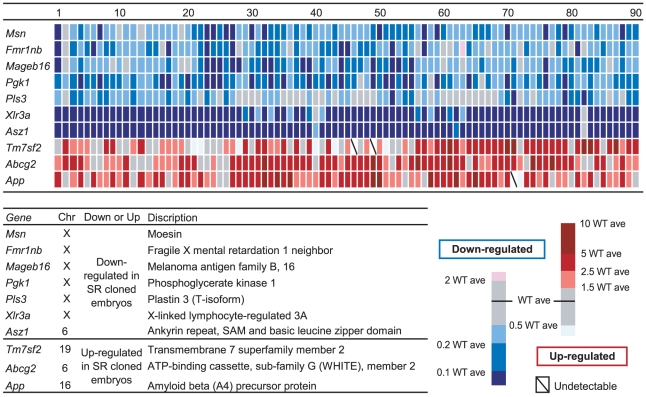
Validation of the Expression Level of Genes Inappropriately reprogrammed in SRCBs. This figure shows expression levels of the 10 genes in the 90 individual SRCBs. The intensity of the blue and red color gradient indicates down- and upregulated expression levels of the genes. The description of the genes tested is presented in the Figure.

Validation of the data obtained by microarray analysis is indispensable for identifying genes differentially expressed in cloned embryos. Here, we carried out a large-scale (90 SRCBs) validation for the expression of 10 genes that were selected as normally expressed in the SR3 embryo (Figure 4). The Bio-Rad iQ5 Multiplex system was used to maximize the number of genes tested using cDNA obtained from each blastocysts. Gene expression in each blastocyst was highly variable, particularly in the cloned embryos. The results, however, clearly indicated that the present microarray method was very efficient for screening the genes differentially expressed in SRCBs. Seven genes, which were identified by the array data as being downregulated, were repressed in most of the cloned embryos. Notably, *Asz1* and *Xlr3a* were expressed at less than 10% of the level observed in the controls in 87 (97%) and 78 (87%) embryos, respectively. Furthermore, in more than 86% of embryos, 4 of the other genes were expressed at less than 50% of the average level observed in the controls. In contrast, the expression levels of 3 genes, *App*, *Abcg2*, and *Tm7sf2*, which were upregulated in the array data, were 1.5-fold higher in 84%, 82%, and 66% of the embryos, respectively, than those in the controls.

### Conclusion

Using credible information from a large-scale transcriptome profiling analysis, we were able to discover some novel properties of mouse SECNT embryos. This is the first overall investigation of SECNT embryos at the blastocyst stage. Our goal was to gain further insight into the reprogramming process and elucidate its underlying mechanisms. We are convinced that our observations can be ascribed to reprogramming occurring in SECNT embryos. Faulty reprogramming at the blastocyst stage would have critical consequences for subsequent differentiation and tissue organization, resulting in large-scale embryo loss at around the implantation stage [1], [19]. This study has revealed that reprogramming differs markedly both between and within the different types of donor cells. Thus, fundamentally, the progress of reprogramming in SECNT is directly affected by the epigenetic status of each donor cell. Our study also showed that there are inappropriately reprogrammed genes common to all SECNT embryos or to each SECNT embryo group. Furthermore, we identified some genes that were inappropriately reprogrammed regardless of donor cell origin. It will be essential to catalogue the epigenetic differences that regulate the expression of these genes because these differences critically affect the fate of the embryos. Studies in this direction may provide valuable insight regarding the epigenetic status, which affects genomic conformation and gene expression. Considering that certain genes were specifically expressed only in surviving embryos, the present results may aid the development of new approaches for selecting these embryos prior to their transfer into recipient females.

## Methods

### Preparation of Recipient Oocytes and Donor Cells

Recipient MII oocytes were collected from mature B6D2F1 (C57BL/6NJcl × DBA/2JJcl) female mice after inducing superovulation in these mice. Donor TT2 ES cells derived from B6CBF1 (C57BL/6/6NJcl × CBA/JNcrlj) embryos were prepared as described in a previous study [20]. The donor SR cells were obtained from 3-day-old male B6CBF1 mice and harvested as described previously [61]. The donor CU cells were collected from ovulated MII oocytes by treatment with hyaluronidase. All mice were maintained and used in accordance with the Guidelines for the Care and Use of Laboratory Animals, as specified by the Japanese Association for Laboratory Animal Science and by the Tokyo University of Agriculture.

### Nuclear Transfer and Culture

SECNT embryos were produced by the injection of a donor nucleus into enucleated oocytes using a piezo-driven system (Prime Tech Ltd., Ibaraki, Japan), using a previously described method [62] and by our standard laboratory method using an inactivated Sendai virus for inducing cell fusion [24], [63]. The activated cloned embryos were cultured in potassium simplex optimization medium (KSOM) at 37°C, under an atmosphere of 5% CO_2_, 5% O_2_, and 90% N_2_ for 4 days. Each blastocyst was lysed in 50 µl of Buffer RLT containing 1% β-mercaptoethanol (Qiagen). Control embryos were obtained from superovulated female C57BL/6NJcl mice, which were mated with male CBA/JNcrlj mice.

### Microarray Analysis

We optimized the manufacturer's protocol with the following modifications for using small quantities of total RNA from samples. Total RNA of each blastocyst was extracted in 11 µl RNase-free water using an RNeasy Micro Kit (Qiagen). The Two-Cycle Eukaryotic Target Labeling Kit (Affymetrix) was used for synthesizing cRNA starting from 9 µl total RNA solutions. The 1^st^ cycle of amplification was conducted in 65 µl reaction mixture. After the quality of the amplified product was verified by Experion™ capillary electrophoresis (Bio-Rad), 10 µg of fragmented cRNA samples was hybridized to a GeneChip® Mouse Genome 430 2.0 Array (Affymetrix, Santa Clara, CA), which contains 45,101 probe sets.

The GeneChip Operating Software (GCOS) version 1.3 (Affymetrix) output files were then loaded into GeneSpring v7.3 (Agilent Technologies, Santa Clara, CA) with per-chip normalization to the 50^th^ percentile and per-gene normalization to the median expression level of the control blastocysts. The boxplots of all signal values for each sample are shown in Figure S3. In the first step of data processing, transcripts with a raw signal intensity greater than 100 for at least one embryo were selected. The filtered genes were used in a one-way ANOVA with the post-hoc test using Tukey's honest significance difference test, and the cut-off value used to identify differentially expressed genes in our study was a false discovery rate of 5%. The genes differentially expressed in each group were saved as lists referred to as SRCB, CUCB, and ESCB gene lists for convenience in further analysis. Principal component analysis (PCA) was employed to analyze the gene expression patterns of all the embryos. Hierarchical clustering was performed with Pearson correlation for measurement of similarity and clustering algorithm with average linkage. The genes were analyzed by gene ontology analysis using FatiGO at Babelomics (www.fatigo.org).

All microarray data is compliant with Minimum Information About a Microarray Experiment (MIAME). The raw data has been deposited in a MIAME-compliant database (DDBJ: http://cibex.nig.ac.jp/index.jsp accession number: CBX109.) The expression report of the present probe sets, the signal (3′/5′) ratio, and the Box plot are shown in Supplemental table S5, Supplemental table S6 and Supplemental Figure S3, respectively.

### Ingenuity Pathway Analysis

The IPA version 7.6 was used to determine the possible biological pathways and the inter-relationships between subsets of differentially expressed genes. A detailed description of the method for performing IPA can be found at www.ingenuity.com. Data sets containing the Affymetrix gene identifiers and their corresponding fold-change in their expression values were uploaded through GeneSpring. Each gene identifier was mapped to its corresponding gene object in the Ingenuity Pathways Knowledge Base. These genes, called focus genes, were overlaid onto a global molecular network developed from information contained in the Ingenuity Pathways Knowledge Base. Networks of these focus genes were then algorithmically generated based on their directly connectivity.

Canonical pathway analysis was used to identify the pathways from the IPA library of canonical pathways that were most significant to the data set. Genes from the data set that were associated with a canonical pathway in the Ingenuity Pathways Knowledge Base were considered for subsequent analysis. The significance of the association between the data set and the canonical pathway was measured in 2 ways. (1) The ratio of the number of genes from the data sets that map to the canonical pathway divided by the total number of genes that map to the pathway was determined. (2) Fischer's exact test was used to calculate the P-value determining the probability that the association between the genes in the dataset and the canonical pathway was explained by chance alone.

### Multiplex Q-polymerase chain reaction

The synthesized cDNA from each blastocyst was employed for quantitative gene expression analysis performed using multiplex real-time quantitative reverse transcriptase polymerase chain reaction (PCR) (Bio-Rad iQ5), which is able to detect expression levels of up to 5 genes in the same well using quantitative PCR probes (Integrated DNA Technologies, Inc.). The detection system using the quantitative PCR probe functions in a manner similar to the TaqMan probe (Applied Biosystems). In this experiment, β-actin was used as the internal control to normalize the target genes. Primer/probe sequences of each gene are shown in Table S7. The samples used for multiplex q-PCR differed from those subjected to microarray analysis.

## Supporting Information

Figure S1 One-way ANOVA post-hoc testing with 5% false discovery rate analysis. Each box shows the number of genes that are statistically similar (green) or different (red) in a group-to-group comparison.
(0.02 MB PDF)

Figure S2 Representative Genes that are Normally Expressed in SR3. The expression levels of Asz1 and Fmr1nb in SR3 ranged from the maximum and minimum for the controls.
(0.02 MB PDF)

Figure S3 Box plot of all signal value for each of the 87 samples. The box whisker plot presents the distribution of the conditions for the active interpretation with respect to the active entity list in the experiment. The box whisker shows the median in the middle of the box, the 25th percentile and the 75th percentile, or the 1st and 3rd quartile. The whiskers are extensions of the box, snapped to the point within 1.5 times the interquartile. The points outside the whiskers are plotted as they are, but in a red color, and could normally be considered the outliers.
(0.10 MB PDF)

Table S1 (0.01 MB PDF)

Table S2 (0.11 MB PDF)

Table S3 (0.12 MB PDF)

Table S4 (0.11 MB PDF)

Table S5 (0.12 MB PDF)

Table S6 (0.06 MB PDF)

Table S7 (0.01 MB PDF)
