# Supplementary material for: Identification of Inappropriately Reprogrammed Genes by Large-Scale Transcriptome Analysis of Individual Cloned Mouse Blastocysts
Source: PLoS One. 2010 Jun 30;5(6):e11274. doi: 10.1371/journal.pone.0011274 (PMC2894852; doi:10.1371/journal.pone.0011274)
Supplement: Figure S1 — One-way ANOVA post-hoc testing with 5% false discovery rate analysis. Each box shows the number of genes that are statistically similar (green) or different (red) in a group-to-group comparison. (0.02 MB PDF) [file pone.0011274.s001.pdf]

	CON	CUCB	SRCB	ESCB
CON	14183	1150	1059	609
CUCB	13033	14183	1070	753
SRCB	13124	13113	14183	649
ESCB	13574	13430	13534	14183

Figure S1.
