# Supplementary figures and images for: Identification of Inappropriately Reprogrammed Genes by Large-Scale Transcriptome Analysis of Individual Cloned Mouse Blastocysts
Source: PLoS One. 2010 Jun 30;5(6):e11274. doi: 10.1371/journal.pone.0011274 (PMC2894852; doi:10.1371/journal.pone.0011274)

Figure S2

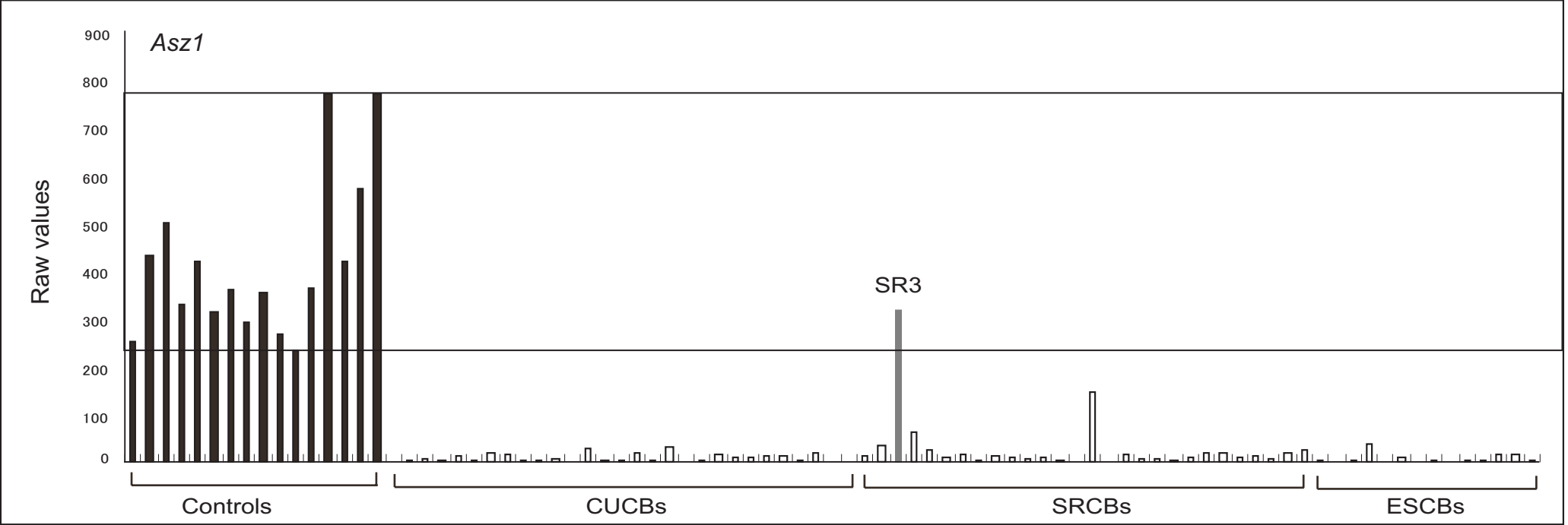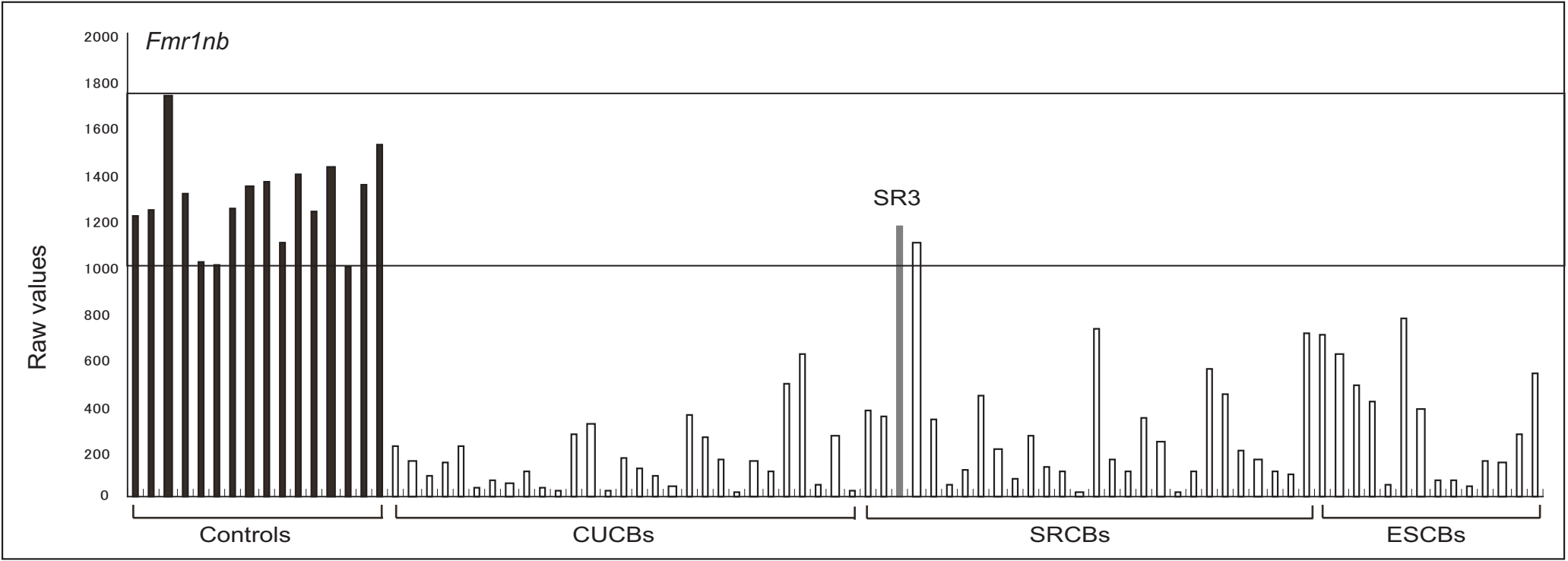

Supplement: Figure S2 — Representative Genes that are Normally Expressed in SR3. The expression levels of Asz1 and Fmr1nb in SR3 ranged from the maximum and minimum for the controls. (0.02 MB PDF) [file pone.0011274.s002.pdf]

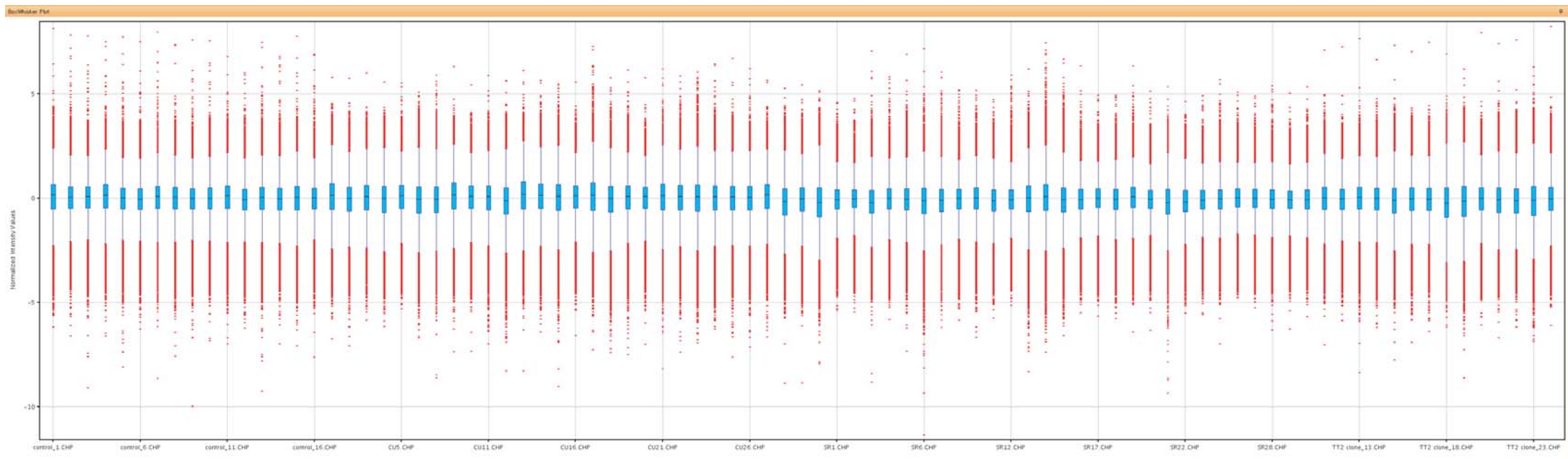

Supplemental Figure S3.

Supplement: Figure S3 — Box plot of all signal value for each of the 87 samples. The box whisker plot presents the distribution of the conditions for the active interpretation with respect to the active entity list in the experiment. The box whisker shows the median in the middle of the box, the 25th percentile and the 75th percentile, or the 1st and 3rd quartile. The whiskers are extensions of the box, snapped to the point within 1.5 times the interquartile. The points outside the whiskers are plotted as they are, but in a red color, and could normally be considered the outliers. (0.10 MB PDF) [file pone.0011274.s003.pdf]
