# Supplementary material for: Identification of Inappropriately Reprogrammed Genes by Large-Scale Transcriptome Analysis of Individual Cloned Mouse Blastocysts
Source: PLoS One. 2010 Jun 30;5(6):e11274. doi: 10.1371/journal.pone.0011274 (PMC2894852; doi:10.1371/journal.pone.0011274)
Supplement: Table S1 — (0.01 MB PDF) [file pone.0011274.s004.pdf]

Supplemental Table S1. Developmental Ability of Embryos cloned from Sertoli cells

No. of activated cloned embryos	No. of 2-cell embryos	No. of morulae and blastocysts	No. of embryos transferred (ET)	No. of implantation sites	No. of pups (%: Pups / ET)
774	523	449	330	126	14(4.2)
