# Supplementary material for: Identification of Inappropriately Reprogrammed Genes by Large-Scale Transcriptome Analysis of Individual Cloned Mouse Blastocysts
Source: PLoS One. 2010 Jun 30;5(6):e11274. doi: 10.1371/journal.pone.0011274 (PMC2894852; doi:10.1371/journal.pone.0011274)
Supplement: Table S2 — (0.11 MB PDF) [file pone.0011274.s005.pdf]

Supplemental Table S2. Descriptions of Canonical Pathways Constructed by Genes Differentially Expressed in Each cloned Group

Order	ALL	CU	SR	ES
1	Role of Oct4 in Mammalian Embryonic Stem Cell Pluripotency	Glycerophospholipid Metabolism	Inositol Metabolism	RhoA Signaling
2	Leukocyte Extravasation Signaling	Amyloid Processing	LXR/RXR Activation	Role of Oct4 in Mammalian Embryonic Stem Cell Pluripotency
3	p53 Signaling	Wnt/β-catenin Signaling	LPS/IL-1 Mediated Inhibition of RXR Function	NRF2-mediated Oxidative Stress Response
4	Nicotinate and Nicotinamide Metabolism	p53 Signaling	Arginine and Proline Metabolism	Type I Diabetes Mellitus Signaling
5	Actin Cytoskeleton Signaling	Tight Junction Signaling	Biosynthesis of Steroids	IL-9 Signaling
6	Fructose and Mannose Metabolism	HER-2 Signaling in Breast Cancer	RAR Activation	Huntington's Disease Signaling
7	Gα12/13 Signaling	Pancreatic Adenocarcinoma Signaling	Aryl Hydrocarbon Receptor Signaling	DNA Methylation and Transcriptional Repression Signaling
8	Riboflavin Metabolism	VDR/RXR Activation	NRF2-mediated Oxidative Stress	IL-8 Signaling
9	Human Embryonic Stem Cell	Interferon Signaling	Calcium Signaling	Type II Diabetes Mellitus Signaling
10	Methionine Metabolism	Glioblastoma Multiforme Signaling	Aminosugars Metabolism	Growth Hormone Signaling
