# Supplementary material for: Identification of Inappropriately Reprogrammed Genes by Large-Scale Transcriptome Analysis of Individual Cloned Mouse Blastocysts
Source: PLoS One. 2010 Jun 30;5(6):e11274. doi: 10.1371/journal.pone.0011274 (PMC2894852; doi:10.1371/journal.pone.0011274)
Supplement: Table S3 — (0.12 MB PDF) [file pone.0011274.s006.pdf]

Table S3. Differentially expressed genes involved in Top GO categories in each donor cell

	Probe ID	[ES](raw)	[SR](raw)	[cu](raw)	Gene Symbol
SR GO:0016126	1423078_a_at	828.8015	195.1738	979.4833	*Sc4mol*
	1451457_at	208.82298	36.02563	403.3878	*Sc5d*
	1460684_at	162.2543	58.25155	263.8276	*Tm7sf2*

	Probe ID	[ES](raw)	[SR](raw)	[cu](raw)	Gene Symbol
ES GO:0050789	1420086_x_at	862.50916	2.546473	5.807298	*Fgf4*
	1416967_at	2277.4438	9.342986	1.119108	*Sox2*
	1460682_s_at	218.20578	2.87745	9.639275	*Ceacam1*
	1418133_at	424.62677	15.12946	135.2874	*Bcl3*
	1449592_at	1031.7913	39.1582	20.26274	*Tcf15*
	1450016_at	1257.0916	84.48518	94.05072	*Ccng1*
	1422912_at	645.9807	48.86761	32.15035	*Bmp4*
	1419018_at	105.98193	10.87192	9.153988	*Rhox6*
	1456225_x_at	467.22113	52.29669	47.49166	*Trib3*
	1424153_s_at	176.4549	24.58283	1.844095	*Sall4*
	1448181_at	177.06918	31.06385	14.48437	*Klf15*
	1448595_a_at	11947.025	2325.285	3470.482	*Bex1*
	1415956_a_at	33.678837	128.1357	220.5989	*Pctk1*
	1449507_a_at	24.303322	93.32291	1159.545	*Cd47*
	1418285_at	12.866414	110.7327	64.43767	*Efnb1*
	1420621_a_at	10.073851	273.2508	468.4097	*App*
	1426397_at	46.449646	436.8082	406.4336	*Tgfbr2*
	1450782_at	2.2248955	64.01466	2235.137	*Wnt4*
	1452670_at	11.661102	368.4554	42.17836	Myl9

	Probe ID	[ES](raw)	[SR](raw)	[cu](raw)	Gene Symbol
CU GO:0016740	1453196_a_at	1.482793	18.78687	256.1661	*Oasl2*
	1418736_at	68.93503	421.559	1947.589	*B3galnt1*
	1460204_at	8.447554	32.86328	165.0034	*Tec*
	1452677_at	8.584272	5.971514	162.2675	*Pnpt1*
	1449630_s_at	35.62468	59.99294	604.5212	*Mark1*
	1434653_at	45.09429	36.63446	575.4872	*Ptk2b*
	1422518_at	71.96353	232.5246	723.6024	*Cask*
	1448183_a_at	28.73729	40.12304	229.875	*Hif1a*
	1419497_at	30.50979	22.68534	189.8535	*Cdkn1b*
	1457046_s_at	31.27565	47.56513	159.0824	*C77370*
	1434061_at	62.68763	64.57081	212.9743	*Rp2h*
	1424638_at	1175.811	96.04173	3695.901	*Cdkn1a*
	1417649_at	79.82433	142.7979	7.506853	*Cdkn1c*
	1449534_at	337.2136	133.6844	28.69917	*Sycp3*
	1420064_s_at	12.89178	182.8024	1	*Tktl1*
	1454138_a_at	114.8099	96.15283	5.625514	*Stk31*
	1456208_at	44.96681	181.6074	1	*A530057A03Rik*
	1434399_at	560.1644	34.75673	5.141852	*Galnt6*
	1430781_at	384.1679	73.43939	3.004887	*Ak7*
