# Supplementary material for: Identification of Inappropriately Reprogrammed Genes by Large-Scale Transcriptome Analysis of Individual Cloned Mouse Blastocysts
Source: PLoS One. 2010 Jun 30;5(6):e11274. doi: 10.1371/journal.pone.0011274 (PMC2894852; doi:10.1371/journal.pone.0011274)

Supplemental Table S4. Coefficients of Correlation between All Samples

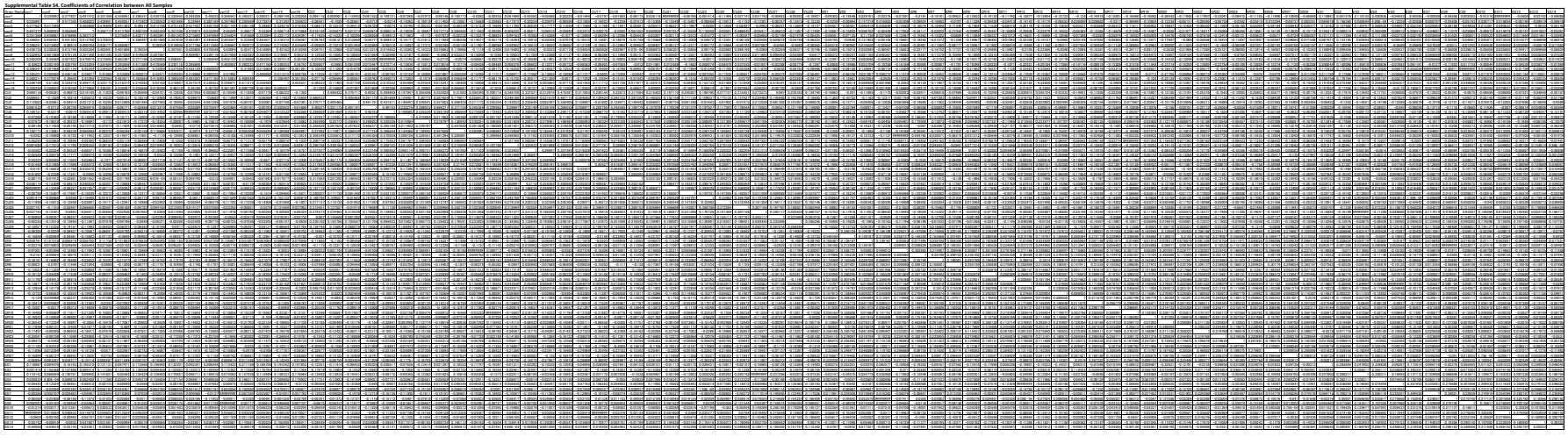

Supplement: Table S4 — (0.11 MB PDF) [file pone.0011274.s007.pdf]
