# Supplementary material for: Identification of Inappropriately Reprogrammed Genes by Large-Scale Transcriptome Analysis of Individual Cloned Mouse Blastocysts
Source: PLoS One. 2010 Jun 30;5(6):e11274. doi: 10.1371/journal.pone.0011274 (PMC2894852; doi:10.1371/journal.pone.0011274)
Supplement: Table S5 — (0.12 MB PDF) [file pone.0011274.s008.pdf]

Supplemental Table S5. Expression Report of Present Probe Sets

Sample Name	Number of present probe sets	P%	Sample Name	Number of present probe sets	P%
Con1	17969	39.8%	SR1	19900	44.1%
Con2	19857	44.0%	SR2	20319	45.1%
Con3	19552	43.4%	SR3	18640	41.3%
Con4	19732	43.8%	SR4	19343	42.9%
Con5	18558	41.1%	SR5	19172	42.5%
Con6	19033	42.2%	SR6	19462	43.2%
Con7	18691	41.4%	SR8	18999	42.1%
Con8	18598	41.2%	SR9	18945	42.0%
Con9	18481	41.0%	SR10	19030	42.2%
Con10	19052	42.2%	SR11	18685	41.4%
Con11	19274	42.7%	SR12	19803	43.9%
Con12	19050	42.2%	SR13	18471	41.0%
Con13	18413	40.8%	SR14	19529	43.3%
Con14	18648	41.3%	SR15	18842	41.8%
Con15	18396	40.8%	SR16	20354	45.1%
Con16	18019	40.0%	SR17	19856	44.0%
			SR18	19010	42.1%
cu1	19154	42.5%	SR19	20065	44.5%
cu2	17507	38.8%	SR20	19116	42.4%
cu3	17775	39.4%	SR21	18184	40.3%
cu4	16905	37.5%	SR22	18128	40.2%
cu5	18597	41.2%	SR23	19612	43.5%
cu6	17738	39.3%	SR25	20426	45.3%
cu7	18074	40.1%	SR26	19840	44.0%
cu8	18548	41.1%	SR27	19833	44.0%
cu9	18342	40.7%	SR28	19526	43.3%
cu10	18107	40.1%	SR29	19306	42.8%
cu11	17438	38.7%	SR30	19738	43.8%
cu12	18363	40.7%			
cu13	19867	44.1%	ES1	19668	43.6%
cu14	18142	40.2%	ES2	19533	43.3%
cu15	18755	41.6%	ES3	18700	41.5%
cu16	18517	41.1%	ES4	18881	41.9%
cu17	17631	39.1%	ES5	17039	37.8%
cu18	18881	41.9%	ES6	18864	41.8%
cu19	19531	43.3%	ES7	17978	39.9%
cu20	18186	40.3%	ES8	15362	34.1%
cu21	18743	41.6%	ES9	15819	35.1%
cu22	18814	41.7%	ES10	18014	39.9%
cu23	19216	42.6%	ES11	17403	38.6%
cu24	19716	43.7%	ES12	17486	38.8%
cu25	18241	40.4%	ES13	15905	35.3%
cu26	18350	40.7%	ES14	18245	40.5%
cu27	15884	35.2%			
cu28	17327	38.4%			
cu29	15440	34.2%			
