# Supplementary material for: Identification of Inappropriately Reprogrammed Genes by Large-Scale Transcriptome Analysis of Individual Cloned Mouse Blastocysts
Source: PLoS One. 2010 Jun 30;5(6):e11274. doi: 10.1371/journal.pone.0011274 (PMC2894852; doi:10.1371/journal.pone.0011274)
Supplement: Table S6 — (0.06 MB PDF) [file pone.0011274.s009.pdf]

Supplemental Table S6. The Signal (3'/5') Ratio of *Gapdh* and *β-action*

SampleName	b-Actin	Gapdh	SampleName	b-Actin	Gapdh
con1	8.7778425	10.611547	SR1	5.74091	8.9943905
con2	11.154052	9.604611	SR2	6.260167	9.734713
con3	7.112683	6.595523	SR3	10.961718	10.421484
con4	7.1224737	6.0176535	SR4	7.241521	9.326849
con5	12.876966	10.608603	SR5	7.823145	12.173708
con6	14.702988	13.06701	SR6	5.9829707	6.6760583
con7	9.311048	10.696405	SR7	4.7655406	6.639841
con8	13.089081	11.860206	SR8	5.8558097	8.982151
con9	12.115263	11.000553	SR9	5.6267014	9.320656
con10	18.333908	14.30989	SR10	10.691717	10.847345
con11	8.154123	9.239252	SR11	9.667199	10.355107
con12	20.455608	14.812883	SR12	6.108022	8.775463
con13	18.995544	14.553926	SR13	5.5074363	9.107455
con14	24.684868	14.486279	SR14	11.137437	12.850852
con15	20.121258	12.670944	SR15	10.224532	15.858984
con16	23.400581	17.837132	SR16	13.3229685	15.651627
CU1	6.6358304	7.296317	SR17	37.287918	24.456747
CU2	12.510183	9.825655	SR18	8.302281	16.496786
CU3	45.889862	24.469658	SR19	11.088065	16.358723
CU4	33.48241	25.854631	SR20	17.52505	16.128714
CU5	37.946888	21.266758	SR21	18.5566	18.24634
CU6	58.406063	17.129503	SR22	11.509092	16.033415
CU7	43.82546	20.106842	SR23	5.6966233	9.999477
CU8	17.231285	27.845434	SR24	9.402284	13.349768
CU9	44.239296	16.90543	SR25	7.071502	13.609827
CU10	32.02931	22.125189	SR26	14.410664	16.575293
CU11	40.862186	23.263882	SR27	9.994475	13.303906
CU12	3.4925234	5.084405	SR28	13.468313	14.6378975
CU13	2.0941882	4.652903	ES1	12.5308	14.018179
CU14	7.1058683	9.756647	ES2	18.518232	15.904053
CU15	7.744314	8.558278	ES3	12.341846	11.972003
CU16	3.5951269	5.9168906	ES4	18.105955	13.055182
CU17	6.04176	7.8505435	ES5	26.738323	12.909072
CU18	7.106329	9.087855	ES6	10.206712	8.402463
CU19	5.5554314	7.377319	ES7	12.629768	11.23893
CU20	15.486058	8.069583	ES8	27.15956	9.731752
CU21	12.462277	7.6880894	ES9	35.249622	13.344854
CU22	6.992665	10.029293	ES10	13.421406	12.280153
CU23	8.560683	7.991734	ES11	23.40748	12.867997
CU24	4.0602574	7.177688	ES12	16.273634	11.334031
CU25	8.699794	9.08098	ES13	20.406864	7.928024
CU26	4.7197247	7.23663	ES14	10.209525	9.060492
CU27	26.610083	9.179198			
CU28	16.604254	9.109906			
CU29	24.00529	10.939458			
