# Supplementary material for: Identification of Inappropriately Reprogrammed Genes by Large-Scale Transcriptome Analysis of Individual Cloned Mouse Blastocysts
Source: PLoS One. 2010 Jun 30;5(6):e11274. doi: 10.1371/journal.pone.0011274 (PMC2894852; doi:10.1371/journal.pone.0011274)
Supplement: Table S7 — (0.01 MB PDF) [file pone.0011274.s010.pdf]

Supplemental Table S7. Primer/Probe Sequence Used for Mq-PCR.

Gene Nai	forward	reverse	probe
Tm7sf2	TAGCTTGGGTACCATTCACCTACA	TAACCAATAGCCTTAAGGAGGCAGAT	/56-TAMN/AGTTCCTGTTGTACCATCCACAGCCTCT/3IAbRQSp/
Pgk1	ATGAGATGATCATTGGTGGTGGAAT	TGACAAAGTCAACAGGCAAGGTAAT	/5TEX615/AAGGTGCTCAACAACATGGAGATTGGCA/3IAbRQSp/
Fmr1nb	TTCAGTAAGCCAGTGTCTGCAATA	AGCTTCAGCAAGAATGGGCAATTT	/5HEX/TTATTCTGGACGCTGGTGATCCTGTTCCGT/3IABlkFQ/
Mageb16	AGAGCATGCTGATTCAAGTTCTACT	ATACAGAAGGAACTGATCTCTGACT	/56-TAMN/TAGTGACACAAGCAGCAACTTCTCTCAGGT/3IAbRQSp/
Asz1	AAATGACCCAACTCATATCCCACTA	TGCAAAGTTAGCTTGCCGATGAAA	/56-FAM/TGAAGAGGTCAGCAGTTACAGTGTGTGGAT/3IABlkFQ/
Pls3	TTGGTCCGATACATGCTTGTTTGTT	ATTTCTGAGACCACATCTCCTTGTT	/56-TAMN/AGGGTGGGCTCAGAATTTAGACGGGAATTT/3IAbRQSp/
Abcg2	TCCTGGCTTCAGTACTTTAGCATT	AAGTGCTGTTGTCCGTTACATTGAA	/5HEX/CCTCGATATGGCTTCACAGCTTTGCAGT/3IABlkFQ/
Msn	AGTTGGAAATGGCTCGAAAGAAGAA	TCTCATCCTGCTCATCATGTTCATT	/56-FAM/AAAGTGAGGCTGTGGAATGGCAGCAAA/3IABlkFQ/
App	AACTGGTGTTCTTTGCTGAAGATGT	ATGACAATCACGGTTGCTATGACA	/56-TAMN/AACAAAGGCGCCATCATCGGACTCAT/3IAbRQSp/
Xlr3a	TCGCAATTACTGGTTAGCACACATT	AGCAGAATGTCAAAGAAAGCCTGTA	/56-FAM/AATTGGAAGGCACACAAGTGGGCTATTGTT/3IABlkFQ/
Actin	GAAATCGTGCGTGACATCAAAG	CTGTGGCATCCATGAAACTACA	/5Cy5/AGGTCATCACTATTGGCAACGAGCGGTT/3IAbRQSp/
